# Discrimination of Oviposition Deterrent Volatile β-Ionone by Odorant-Binding Proteins 1 and 4 in the Whitefly *Bemisia tabaci*

**DOI:** 10.3390/biom9100563

**Published:** 2019-10-03

**Authors:** Fengqi Li, Du Li, Youssef Dewer, Cheng Qu, Zhen Yang, Jiahui Tian, Chen Luo

**Affiliations:** 1 Beijing Key Laboratory of Environment Friendly Management on Fruit Diseases and Pests in North China, Institute of Plant and Environment Protection, Beijing Academy of Agriculture and Forestry Sciences, Beijing 100097, China; pandit@163.com (F.L.); lidu94@163.com (D.L.); quch1990@sina.com (C.Q.); tianjh0920@163.com (J.T.); 2 Hubei Key Laboratory of Insect Resource Application and Sustainable Pest Control, College of Plant Science and Technology, Huazhong Agricultural University, Wuhan 430070, China; 3 Bioassay Research Department, Central Agricultural Pesticide Laboratory, Sabahia Plant Protection Research Station, Agricultural Research Center, Alexandria 21616, Egypt; dewer72@yahoo.com; 4 Chinese Materia Medica College, Tianjin University of Traditional Chinese Medicine, Tianjin 300000, China; yzwygb@126.com

**Keywords:** *Bemisia tabaci*, odorant-binding protein, competitive binding, β-ionone

## Abstract

The whitefly, *Bemisia tabaci*, is an important invasive economic pest of agricultural crops worldwide. β-ionone has a significant oviposition repellent effect against *B. tabaci*, but the olfactory molecular mechanism of this insect for recognizing β-ionone is unclear. To clarify the binding properties of odorant-binding proteins (OBPs) with β-ionone, we performed gene cloning, evolution analysis, bacterial expression, fluorescence competitive binding assay, and molecular docking to study the binding function of OBP1 and OBP4 on β-ionone. The results showed that after the OBP1 and OBP4 proteins were recombined, the compound β-ionone exhibited a reduction in the fluorescence binding affinity to <50%, with a dissociation constant of 5.15 and 3.62 μM for OBP1 and OBP4, respectively. Our data indicate that β-ionone has high affinity for OBP1 and OBP4, which play a crucial role in the identification of oviposition sites in *B. tabaci*. The findings of this study suggest that whiteflies employ β-ionone compound in the selection of the suitable egg-laying sites on host plants during the oviposition behavior.

## 1. Introduction

Insects have many chemosensory organs, including antennae and other parts of the body, that play a key role in communication, such as in host-plant recognition. These chemosensory organs enable insects to successfully complete a series of important functions such as foraging, mating, oviposition, and avoidance. These behaviors contribute to insect survival in a complex environment [1,2]. Various types of proteins are produced by insect chemosensory organs, which include odorant-binding proteins (OBPs), chemosensory proteins (CSPs), olfactory receptors [3], odorant-degrading enzymes (ODES), and sensory neuron membrane proteins (SNMPs). OBPs are the most extensively studied chemosensory proteins. The first OBP was discovered in the 1980s in the male moth antenna of *Antheraea polyphemus* [3], which then subsequently prompted entomologists to study OBP function. OBPs allow chemical information exchange between insects and the environment [4]. OBPs are abundantly expressed in insect chemosensory organs and taste organs [5,6], and their main function is to bind and transport odorant molecules [7]. OBPs have been a research hotspot in insect molecular biology in recent years.

*Bemisia tabaci* (Gennadius) is an important agro-forest invasive pest and described as a species complex, of which types B and Q (Middle East-Asia Minor1 cryptic species and Mediterranean cryptic species) are highly invasive species, occur most commonly, and are the most serious and potentially devastating types. Since the 1980s, with the importation of flowers and other seedlings, B-type *B. tabaci* has further spread around the world, and has become a serious global problem, with annual economic losses exceeding USD 300 million [8]. Q-type *B. tabaci* was first detected in China in 2003 and gradually supplanted the B-type, which had been ubiquitous in China [9,10]. *B. tabaci* has a wide range of hosts, strong viability, large egg production, rapid population growth, and rapid development of resistance to insecticides [11]. Due to the increasing harm of *B. tabaci* and its economic importance, it is very important to study alternative safe strategies and methods for the effective control of *B. tabaci*. Currently, the control of *B. tabaci* still relies mainly on pesticides, especially when large-scale outbreaks of *B. tabaci* occur, of which pesticides are almost the only means of prevention. The extensive use of pesticides has not only caused severe environmental pollution but has also a great impact on natural enemies and bees [12,13]. Moreover, *B. tabaci* has developed resistance to a diverse group of pesticides after their continuous application in the field [14]. To date, *B. tabaci* has shown resistance to more than 40 active ingredients of insecticides [15]. Therefore, it is imperative to explore and develop new tools of non-toxic strategies for controlling whitefly in integrated pest management.

The host plants of *B. tabaci* are widely and conservatively estimated to have more than 500 species [16], including important crops such as cotton, tobacco, vegetables, and flowers. Despite its wide range of host species, *B. tabaci* is capable of selecting suitable host plants to locate feeding and oviposition sites [17,18]. This selective oviposition behavior can be mainly attributed to differences in plant-host volatiles. It is thus generally believed that *B. tabaci* has the ability to identify and distinguish different hosts through its chemosensory system for oviposition behavior.

**Figure 1:**
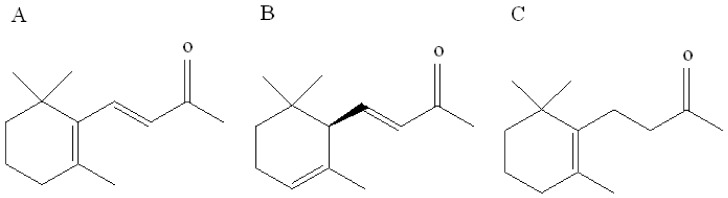
Chemical structures of (**A**) β-ionone, (**B**) α-ionone, and (**C**) hydroxy-β-ionone.

*β*-ionone is a volatile aromatic component contributor to the aroma of roses that is naturally present in various plants such as cotton and tomato. In plants, the biosynthesis of β-ionone is controlled by the carotenoid cleavage dioxygenase gene, which converts β-carotene to β-ionone in vivo and is a key gene controlling *β*-ionone biosynthesis [19]. In addition, two analogs of *β*-ionone, namely, α-ionone and dihydro-β-ionone, also commonly occur as plant volatiles and play a key role in the interaction between plants and herbivores. These three compounds exhibit similar chemical structures that are derived from a 2,6,6-trimethylcyclohexyl carbon skeleton and a butyl side-chain situated at position C-1 and harboring a carbonyl group. Isomers α-ionone and β-ionone differ in terms of the position of the endocyclic double bond, i.e., C-2 position of the ring in α-ionone, whereas it is C-1 in *β*-ionone. The structure of dihydro-*β*-ionone is similar to that of β-ionone except for the exocyclic C-3 double bond located on the alkyl side-chain (Figure 1). Cáceres et al. [10] overexpressed the carotenoid cleavage dioxygenase1 (*CCD1*) gene in *Arabidopsis thaliana*. The gene *CCD1* encodes the CCD1 enzyme that cleaves a broad range of carotenoids to generate aldehydes and ketones. Volatile collection followed by gas chromatography-mass spectrometry detection indicated a significant increase in the content of β-ionone in overexpressing plants. Further investigation using overexpressing plants and *β*-ionone standards revealed that *β*-ionone at concentrations of 0.1 ng/μL and 0.5 ng/μL can significantly inhibit *B. tabaci* egg production. This study revealed that β-ionone can be considered as an important oviposition repellent agent against *B. tabaci*. However, the olfactory molecular mechanism for recognition of this compound in *B. tabaci* is still unclear. We have recently identified several OBPs from *B. tabaci* by transcriptome analysis [20]. However, the function of these OBPs is largely unknown. Therefore, the current study was aimed to investigate the binding characteristics of two *B. tabaci* OBPs (OBP1 and 4) with *β*-ionone to elucidate the molecular mechanism underlying *β*-ionone recognition. It will provide a better understanding of host preferences and repellent effects against *B. tabaci*, as well as potential schemes in controlling further spread of this invasive pest.

## 2. Materials and Methods

### 2.1. Insect Rearing

The *B. tabaci* Q type was established in the greenhouse of the Beijing Academy of Agricultural and Forestry Sciences (Beijing, China). The host plant was the tomato variety Xianke 8. The feeding conditions include a photoperiod light:dark = 16:8 h, temperature 25–28 °C, and relative humidity ranging from 60 to 80%.

### 2.2. Cloning and Sequence Analysis of OBP1 and OBP4

Total RNA was extracted from about 200 heads of whitefly using TRIzol (ThermoFisher Scientific-Life Technologies, Carlsbad, CA, USA). The first strand cDNA was synthesized using a PrimeScript RT reagent kit (TaKaRa, Dalian, China) with a gDNA Eraser, 2 μL of DNA eraser buffer, 1 μL of gDNA eraser, 1 μg of RNA, RNase-free dH_2_O to 10 μL, 42 °C for 2 min, then placed on ice, plus 1 μL of PrimeScript RT enzyme mix, 1 μL of RT primer mix, 4 μL of PrimerScript buffer, and 4 μL RNase-free dH_2_O. The reaction conditions were as follows: 37 °C for 15 min and 85 °C for 5 s. The synthesized cDNA was stored at –20 °C until use.

According to our previously reported OBP1 and OBP4 sequences [9] (GenBank accession number: KY305457 and KY305460, respectively), signal peptide predictions were performed using SignalP [21]. The sequence primers were designed using PrimerPremier 5 (PREMIER Biosoft, Palo Alto, CA, USA), and *Nde*I and *Xho*I restriction sites were added (underlined). The primers were as following: OBP1 forward: 5′-GGAATTCCATATGGTTACCCATGCTGAAATGGAC-3′; OBP1 reverse: 5′-CCGCTCGAGATTGAACCAGCCAAGCTCC-3′, OBP4 forward: 5′-GGAATTCCATATGCAAGACGCGAAGCAAAAAG-3′; OBP4 reverse: 5′-CCGCTCGAGAGCCTTGATGTAAAAGTTGTGC-3′.

Each PCR reaction consisted of the following: 10 μL of the 5× PrimeSTAR Buffer, 4 μL of the dNTP mixture, 1 μL of each of the forward and reverse primers, 0.5 μL of cDNA, 0.5 μL of PrimeSTAR HS DNA polymerase (TaKaRa), and 33 μL of ddH_2_O. The PCR program was an initial denaturation at 94 °C for 3 min; followed by 30 cycles of 94 °C for 15 s, 60 °C for 5 s, and 72 °C for 1 min; then 72 °C for 10 min. The PCR products were detected by 1% agarose gel electrophoresis, and the target fragment was recovered and ligated into a pMD18-T cloning vector (TaKaRa, Dalian, China) and identified by PCR.

OBP amino acid sequences were aligned using ClustalX version 1.83 [22]. Phylogenetic analysis was performed with MEGA5 software [23] using the maximum likelihood method [23], heuristic searches were conducted using nearest neighbor interchange, and bootstrap was set as 1000 replicates. Similar amino acid sequences of Q-type *B. tabaci* OBP1 and OBP4 were detected using the National Center for Biotechnology Information (NCBI) BLAST, and the 28 OBP from 22 different insects were used for phylogenetic analysis; the protein name and NCBI accession numbers are listed in Table S1. Btab_Q_OBP1 and Btab_Q_OBP4 from Q-type *Bemisia tabaci* and Btab_B_OBP1 and Btab_B_OBP4 from B-type *Bemisia tabaci* were included in the study. The 4 OBPs with 9 OBPs binding β-ionone were used for motif analysis [24,25,26,27,28,29,30]. The Multiple EM for Motif Elicitation (MEME) online server [31] was used to discover and analyze the motifs [32]. Parameters used in the motif predictions of this study were: minimum width = 6, maximum = 10, maximum number of motifs to find = 8. Species abbreviations were as follows: Apis, *Acyrthosiphon pisum*; Mvic, *Megoura viciae*; Dvit, *Daktulosphaira vitifoliae*; Bbra, *Brevicoryne brassicae*; Ebal, *Episyrphus balteatus*; Haxy, *Harmonia axyridis*; Dcit, *Diaphorina citri*; Nlug, *Nilaparvata lugens*; Agos, *Aphis gossypii*; Lstr, *Laodelphax striatella*; Psal, *Pterocomma salicis*; Agly, *Aphis glycines*; Acra, *Aphis craccivora*; Hobl, *Holotrichia oblita*; Hele, *Hylamorpha elegans*; Lsti, *Loxostege sticticalis*; Csup, *Chilo suppressalis*; Acer, *Apis cerana*; Alin, *Adelphocoris lineolatus*; and Mmed, *Microplitis mediator*.

### 2.3. Bacterial Expression and Protein Purification of Recombinant OBP1 and OBP4

The positive clones of OBP1 and OBP4 genes were digested with *Nde*I and *Xho*I simultaneously with a pET-30a (+) expression vector, ligated with T4 DNA ligase, and transformed into Trans 5α competent cells. After PCR and double restriction enzyme digestion, the positive plasmids were transformed into *Escherichia coli* BL21 (DE3) competent cells. The positive clones were selected for culture to an OD_600_ value of 0.4−0.6, and isopropyl-β-D-thiogalactopyranoside (IPTG) was added at a final concentration of 1 mmol·L^−1^. Protein expression was induced at 28 °C and 180 rpm for 10 h. The cells were collected by centrifugation, and the bacterial lysate was added for ultrasonication (sonicated for 30 min, worked for 3 s, stopped for 5 s). After crushing, the supernatant and the precipitate were collected by centrifugation, and protein expression was detected by SDS-PAGE.

The protein supernatant was added to a HisTrap affinity chromatography column (TaKaRa, Dalian, China) and purified by gradient imidazole buffer (0, 20, 50, 100, 250, 500 mmol·L^−1^), and the target protein eluate was eluted at 50 mM Tris-HCl (pH 7.4) and dialyzed for 24 h. Protein detection was performed by SDS-PAGE, and protein concentrations were measured according to instructions of the bicinchoninic acid (BCA) Protein Assay Kit (ThermoFisher Scientific-Life Technologies, Carlsbad, CA, USA).

### 2.4. Ligand Binding Experiments of OBP1 and OBP4

*N*-Phenyl-1-naphthylamine (1-NPN), 4,4′-Dianilino-1,1′-binaphthyl-5,5′-disulfonic acid dipotassium salt (*bis*-ANS), β-ionone, α-ionone, and hydroxy-β-ionone were diluted with chromatographic-grade methanol to a final concentration of 1 mmol·L^−1^, and the protein was diluted with 50 mM Tris-HCl (pH 7.4) to a final concentration of 2 μmol·L^−1^. When 1-NPN was used as fluorescent probe, the excitation wavelength of the fluorescence spectrophotometer was set to 337 nm, and the scanning range was 350−500 nm. When *bis*-ANS was used as fluorescent probe [33,34], the excitation wavelength of the fluorescence spectrophotometer was set to 295 nm, and the scanning range was 300−550 nm. The fluorescent probe was gradually added to the protein-containing quartz tube to make it close to saturation. According to the Scatchard equation [35], the binding dissociation constants K_1-NPN_ of OBP1 and the fluorescent probe 1-NPN and K_bis-ANS_ of OBP4 and fluorescent probe bis-ANS were determined. Protein and fluorescent probes were added to the quartz dish (the final concentration was 2 μM), and ionone was gradually added to determine the binding ability of the protein and ionone, and the dissociation constant K_i_ was calculated using the formula: K_i_ = [IC_50_]/(1 + [L]/K_L_), where [IC_50_] is the concentration of the ligand at which the fluorescence intensity is reduced by half, [L] is the concentration of the free fluorescent probe, and K_L_ is the binding dissociation constant of the protein and the fluorescent probe. N-Phenyl-1-naphthylamine (1-NPN, Chemical Abstract Service (CAS): 90-30-2), 4,4’-dianilino-1,1’-binaphthyl-5,5’-disulfonic acid dipotassium salt (*bis*-ANS, CAS: 65664-81-5), methanol (CAS: 67-56-1), α-ionone (CAS: 127-41-3), β-ionone (CAS: 14901-07-6), and dihydro-β-ionone (CAS: 17283-81-7) were purchased from Sigma-Aldrich (St. Louis, MO, USA).

### 2.5. Molecular Docking

After searching the current Protein Data Bank (PDB) (http://www.rcsb.org) using the amino acid sequence of OBP1 and OBP4 as a probe, the crystalline structures of a vetch aphid *Megoura viciae* OBP3 (PDB ID: 4Z39, Chain A, resolution 1.3 Å) [36] and currant-lettuce aphid *Nasonovia ribisnigri* OBP3 (PDB ID: 4Z45, Chain A, resolution 2.02 Å) [36] were selected as templates. Sequence alignment of the target and template were then conducted, and three initial models were built using MODELLER [37]. Ramachandran plot and Profile-3D [23] was used to estimate the structural alignment of the models. Ramachandran plot was generated by ProCheck [38]. Protein preparation were conducted using the Clean Protein module to correct problems such as incomplete residues, alternate conformations and modified hydrogens. In addition, the small molecule was prepared using the minimize ligand module. CDOCKER was used to analyze the interaction energy between OBP and β-ionone based on CHARMm [37]. All analyses were conducted in Discovery Studio (Accelrys, San Diego, CA, USA) version 4.0.

## 3. Results

### 3.1. Gene Cloning and Sequence Analysis

**Figure 2:**
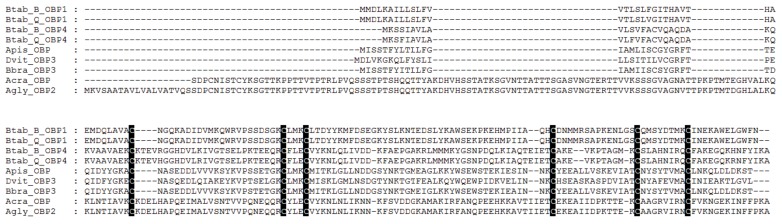
Sequence alignment of OBPs.

After total RNA extraction, cDNA synthesis, PCR amplification, and sequencing, the OBP1 and OBP4 gene sequences of *B. tabaci* were obtained, which were consistent with the OBP sequences in our previous reports. The open reading frame (ORF) is 429 bp in length, with predicted signal peptides consisting of 19 and 24 amino acids starting from the N-terminus. *B. tabaci* OBP1 and OBP4 have six conserved cysteine sites (Figure 2).

**Figure 3:**
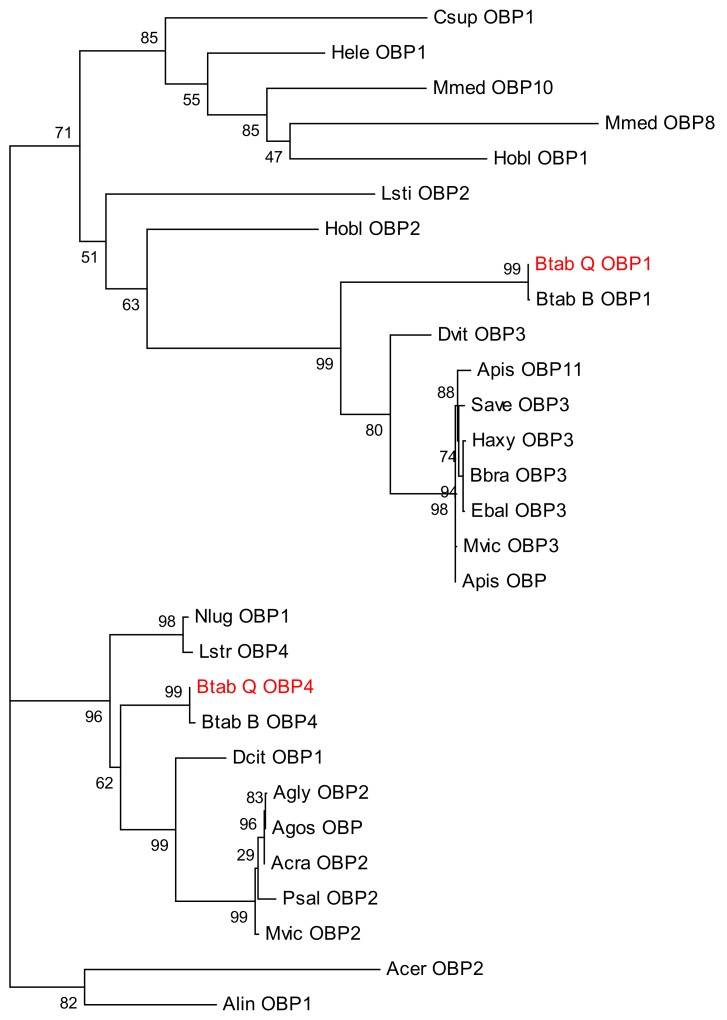
Phylogenetic tree of odorant-binding protein 1 (OBP1), OBP4, and other insect OBPs.

**Figure 4:**
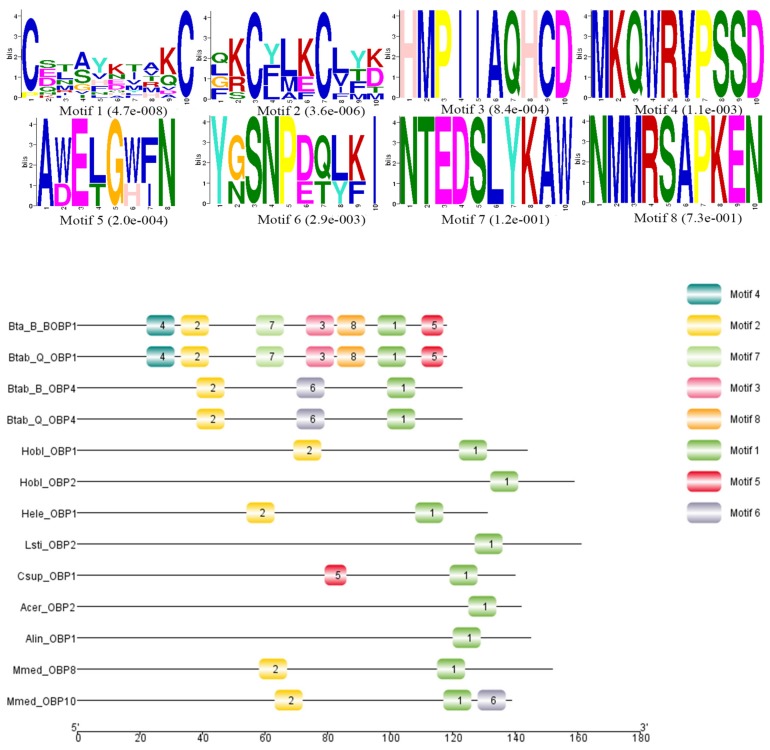
Motif analysis of OBPs binding β-ionone. The upper images list the eight motifs discovered in the 13 OBPs using the MEME (version 5.0.5) server [31] with the following parameters: minimum width = 6, maximum width = 10, maximum number of motifs to find = 8. The lower part of the figure indicates approximate locations of each motif on the protein sequence. The numbers in the boxes correspond to the numbered motifs in the upper part of the figure. The numbers on the bottom show the approximate locations of each motif on the protein sequence, starting from the N-terminal. The protein names and NCBI accession numbers of these OBPs are listed in Table S1.

Sequence alignment and phylogenetic tree construction of *B. tabaci* OBP1, OBP4, and the OBPs of other insects show that Q-type *B. tabaci* OBP1 is closely related to B-type *B. tabaci* OBP1. The protein sequence identity of OBP1 between B and Q species was as high as 99%, with a score of 292, and an *E*-value of 2 × 10^−85^. OBP4 has a sequence identity of 96% between the two species, a score of 275, and an *E*-value of 7 × 10^−79^. Based on the phylogenetic tree, Q-type *B. tabaci* OBP4 and B-type *B. tabaci* OBP4 showed the closest phylogenetic relationship (Figure 3). *B. tabaci* OBP1 and LstiOBP2 from *Loxostege sticticalis*, HoblOBP2 from *Holotrichia oblita* in a large clan, and LstiOBP2, HoblOBP2 have been confirmed to bind β-ionone. In total, eight conservative motifs were found after MEME motif analysis (Figure 4). Among 13 OBPs binding β-ionone, only motif1 exists in all OBP genes, and HoblOBP2, LstiOBP2, AcerOBP2 and AlinOBP1 only have motif1. This result indicates that motif1 may play an important role in binding to β-ionone. 

### 3.2. Binding Characteristics of OBP1 and OBP4

The recombinant plasmids of pET30a-OBP1 and pET30a-OBP4 were constructed and transformed into BL21 (DE3) competent cells, and protein expression was induced at 1 mmol·L^−1^ IPTG, 28 °C, and 180 rpm. The target protein band was obtained after passing through a Ni-NTA column (Figure S1), and protein concentration was measured using the BCA method [21] after dialysis for 24 h.

**Figure 5:**
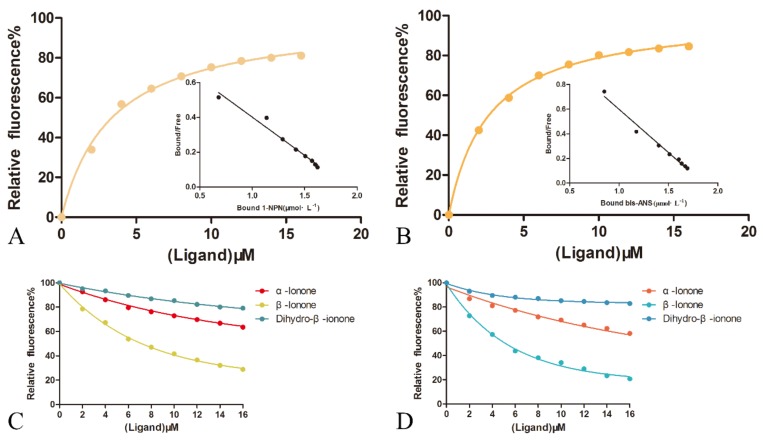
Ligand-binding test of OBP1 and OBP4 to ionone. (**A**) Binding of OBP1 and 1-NPN; (**B**) binding of OBP4 and *bis*-ANS; (**C**) competitive combining of ionone with 1-NPN and OBP1 protein; and (**D**) competitive binding of ionone with *bis*-ANS and OBP4 protein.

The binding characteristics of OBP1 and fluorescent probe 1-NPN were detected by molecular fluorescence spectrometry (Figure 5A).

The binding curve was linearized using the Scatchard equation and Y = −0.4389X + 1.913 (*R*^2^ = 0.9701). The dissociation constant is 3.379 μmol·L^−1^, indicating that the 1-NPN probe can be used in subsequent fluorescence competition binding assays. Using the same method, we used molecular fluorescence spectrometry to detect the binding characteristics of OBP4 and fluorescent probe *bis*-ANS (Figure 5B). The binding curve was linearized using the Scatchard equation and Y = −0.7082X + 1.851 (R^2^ = 0.9817). The dissociation constant was 2.675 μmol·L^−1^, indicating that the *bis*-ANS probe can be used for subsequent fluorescence competition binding experimental studies. We also detected the binding characteristics of *bis*-ANS for OBP1 and 1-NPN for OBP4, but the linear regressions were far from ideal (*bis*-ANS-OBP1: Y = −0.1975X + 0.4881, *R*^2^ = 0.4573; 1-NPN-obp4: y = −0.4819x + 0.8533, *R*^2^ = 0.4824) (Figure S2A,B).

β-ionone was added to a mixture of OBP1 and 1-NPN at a concentration of 2 μM, and a fluorescence competition binding experiment was performed. The fluorescence intensity was changed with the concentration of the ligand and the IC_50_ was calculated, and the dissociation constant K_i_ was calculated according to the formula (Table S2). The β-ionone was tested to compete for fluorescence intensity below half of the initial value (Figure 5C), with IC_50_ of 7.16 μM and a dissociation constant K_i_ of 5.15 μM. In the same manner, β-ionone was added to a mixture of OBP4 and *bis*-ANS at a concentration of 2 μM to perform a fluorescence competition binding experiment, changes in fluorescence intensity based on ligand concentration were recorded, IC_50_ was calculated, and the dissociation constant K_i_ was calculated according to the formula. After testing, β-ionone was able to compete for fluorescence intensity below half of the initial value (Figure 5D), with IC_50_ of 5.14 μM and a dissociation constant K_i_ of 3.62 μM. We also tested the binding ability of two analogs of β-ionone, including α-ionone and dihydro-β-ionone, to OBP1 and OBP4. α-Ionone showed weaker binding to both OBP1 and OBP4. Dihydro-β-ionone hardly displaced the fluorescent probe from both complexes. Though the linear regressions of *bis*-ANS with OBP1 and 1-NPN with OBP4 were far from ideal, we used them as fluorescent probes, respectively, to test the binding ability of ionone to OBP1 and OBP4. β-ionone bonded well with OBP1 and OBP4, while α-ionone and dihydro-β-ionone did not (Figure S2C,D).

### 3.3. Homology Modeling and Molecular Docking

**Figure 6:**
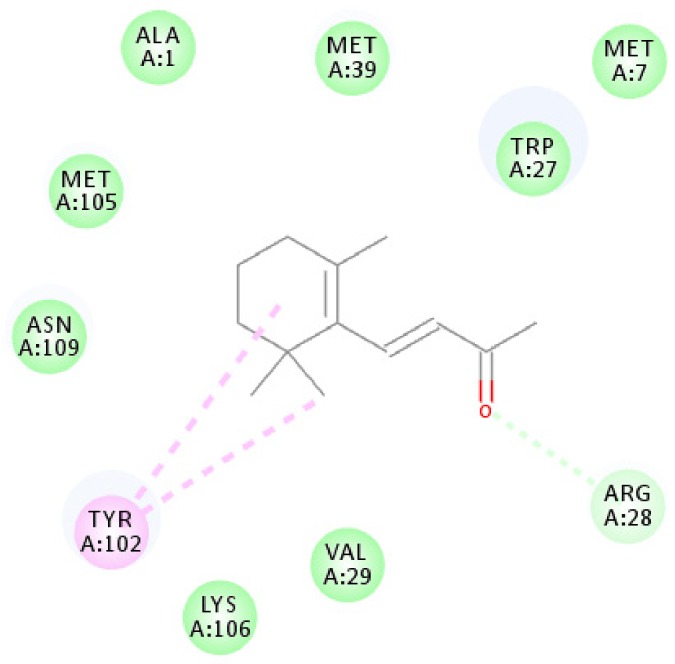
Molecular docking schematic of OBP1 to β-ionone. Diagram of the interactions of β-ionone with key binding site residues. Residues indicated in the figure have a distance to β-ionone of <0.1 Å. Pi-alkyl and van der Waals interactions are indicated in pink and green dashed lines, respectively.

Using homology modeling, OBP1 shared 35% and 34% sequence identity with templates 4Z39 and 4Z45 (Figure S3). However, for OBP4, no template shared a sequence similarity of >30%, and thus, we only used OBP1 for homology modeling and molecular docking analysis. Based on the results of the Ramachandran plot (Figure S4), 96.3% of all residues were in the favored regions, with no residues in disallowed regions. In addition, the verified score of the OBP1 model using 3D-Profile was 43.12, with verified expected high and low scores of 53.29 and 23.92, respectively. CDOCKER analysis indicated an interaction energy of 19.2217 kcal/mol, and the 2D ligand interaction diagram is shown in Figure 6.

Figure 6 shows that the β-ionone molecule is surrounded by ALA1, MET7, TRP27, ARG28, VAL29, MET39, TYR102, MET105, LYS106, and ASN109, which are potential active amino acids for the OBP1-β-ionone interaction. The amino acid TYR102 was suitable for the formation of alkyl interactions, and nine other amino acids form internal energy using van der Waals potentials.

## 4. Discussion

During evolution, insects have adapted a sensitive chemosensory system. In the past 30 years, entomologists have conducted in-depth research studies on the molecular mechanisms of insect sensing of host plant volatiles and sex pheromones, and determined that sensitive chemical systems play an important role in insect searches for hosts, mating, and oviposition [39,40,41]. Thus, elucidating how whitefly recognizes oviposition repellent volatiles may provide a theoretical basis for the ecological control of pests.

Plant volatiles play a very important role in the chemical communication of insects, and these volatiles play a key role in influencing the selection of insects for feeding and ovipositioning on hosts. In this study, we analyzed the binding properties of *B. tabaci* odorant-binding proteins OBP1 and OBP4 to the ovipositioning repellent volatile β-ionone. The respective dissociation constants of β-ionone and OBP1 and OBP4 were 5.15 μmol/L and 3.62 μmol/L; both were <10 μmol/L, indicating strong binding ability. β-ionone is a floral odorant of lipids, and insects use some floral volatiles to locate plants [42]. The results of this study are consistent with the findings of previous studies [24,25,26,27,28,29,30]. Some insect OBPs strongly bind to β-ionone in test candidate substances, indicating that β-ionone has high affinity for insect OBPs. Because β-ionone is a floral odor chemical and is widely found in flowering plants, it is possible that OBP1 and OBP4 are involved in insect ovipositioning on host flowering plants. Motif analysis showed that motif1 existed in all the OBPs, and this conserved domain may play an essential role for the OBPs to bind β-ionone. In subsequent studies, the experiment should be further confirmed to confirm the role of this motif.

In addition, previous studies involving bioinformatic analysis of the genome and transcriptome of *B. tabaci* have identified eight OBP-encoding genes in *B. tabaci* [9,31], among which OBP8 has strong binding affinity to β-ionone (k_i_ value was 13.32 μmol/L). This indicates that many insect olfactory proteins, including OBP in whitefly, function in recognizing β-ionone. Our past expression profiling studies have also shown that OBP1 and OBP4 are highly expressed in heads of female adults [20]. Accordingly, we can infer that β-ionone may play a crucial role in the selection of oviposition hosts for *B. tabaci*. Therefore, multiple olfactory proteins of *B. tabaci* retain the function of binding this compound. In addition, OBP2 and OBP6 were also highly expressed in the heads of female adults [20], and the binding characteristics of these two OBPs to β-ionone need to be further confirmed by experiments in the future.

In addition, in this study, 1-NPN was found to be a suitable fluorescent reporter for OBP1, and *bis*-ANS is a suitable fluorescent reporter for *B. tabaci* OBP4. We found that the commonly used 1-NPN probe is not suitable as a fluorescence reporter for OBP4. These results indicate that in the functional study of the olfactory proteins of *B. tabaci*, researchers should exercise more caution in selecting fluorescent reporters, and different olfactory proteins may be required for various fluorescent reporters such as 1-NPN or *bis*-ANS.

In the current study, α-ionone showed a weak binding to OBP1 and OBP4; according to previous study, a-ionone also showed a weak ovipositioning repellent to whitefly at the doses of 0.05 ng/μL and 0.5 ng/μL [10]. Thus, this binding ability is consistent with ovipositioning repellent activity, further emphasizing the importance of OBP1 and OBP4 in the binding of ovipositioning repellent compounds.

Protein homology and evolutionary analyses revealed that OBP1 and OBP4 have extremely high homology (99% and 96%, respectively) between *B. tabaci* B and Q, thereby indicating orthology, prompting us to hypothesize that OBP1 and OBP4 in B-type whitefly also function in β-ionone recognition. In addition, OBP3 of *Daktulosphaira vitifoliae* is on the same branch of the phylogenetic tree and, thus, we can infer that the gene also functions in β-ionone recognition. The ability of these candidate OBP genes to bind to β-ionone thus needs to be confirmed experimentally in future studies.

In this study, modeling of OBP1 structures and their docking relationship with β-ionone molecules can be used to predict the oviposition repellent compounds of *B. tabaci* by molecular docking to other small molecules. In addition, it can also be used to promote modifications of β-ionone by chemical modification to develop the analog of β-ionone. Similar studies have been successfully conducted in the development and application of aphid alarm pheromone (E)-β-farnesene analogs, which provides a good case for the further field application of β-ionone in whitefly management. For OBP4, because the homology with the PDB database template is <30%, it is not suitable for structural modeling research studies [38,43]. The availability of additional crystal structures of insect OBPs may improve our understanding of the interaction between OBP4 and β-ionone.

β-Ionone is a common plant aroma chemical, and previous studies have found that the compound can attract bees to pollinate and increase crop yield. Caceres et al. [10] found that β-ionone has a strong repellent effect toward both the flea beetle and the spider mite, and significant oviposition deterrence to whiteflies. Therefore, the compound has potential applications to agro-ecosystems. For example, in the tomato production system, the compound can be formulated as a molecule agent for spraying on tomato, thereby improving bee pollination and affecting the oviposition of whiteflies, and reaching the goals of high-quality, high-yield, and safe tomato production.

The current study explored the olfactory molecular mechanism of β-ionone in *B. tabaci* using OBP. In the future, it is necessary to comprehensively identify the odorant receptor and neurons in whitefly that contribute to β-ionone recognition to fully reveal the underlying olfactory molecular mechanism and provide valuable clues for the prevention and control of this insect pest. The study on the function of OBPs of *B. tabaci* may help in elucidating the underlying host preference mechanism of *B. tabaci*, provide a theoretical basis for its ecological control, and promote the application of “push-pull strategy” in the field or greenhouse.

## 5. Conclusions

We tested the binding function of OBP1 and OBP4 proteins with β-ionone, which is an oviposition repellent volatile against *B. tabaci*. Both proteins showed high affinity for β-ionone, with dissociation constants with OBP1 and OBP4 of 5.15 and 3.62 μM, respectively. These proteins play an important role in the identification of oviposition volatiles in *B. tabaci*.

## Supplementary Materials

The following are available online at https://www.mdpi.com/2218-273X/9/10/563/s1, Figure S1: Expression and purification of the recombinant OBP1 and OBP4 protein. Figure S2: Ligand-binding test of OBP1 and OBP4 to ionone. Figure S3: Homology alignment of OBP1 with temple. Figure S4: Ramachandran plot for OBP1. Table S1: Accession numbers for amino acid sequences of OBPs in phylogenetic tree. Table S2: Binding affinities of odorants to OBP1 and OBP4.

## References

[1] Robertson H.M., Warr C.G., Carlson J.R. (2003). Molecular evolution of the insect chemoreceptor gene superfamily in *Drosophila melanogaster*. Proc. Natl. Acad. Sci. USA.

[2] Swanson J.A., Torto B., Kells S.A., Mesce K.A., Tumlinson J.H., Spivak M. (2009). Odorants that induce hygienic behavior in honeybees: Identification of volatile compounds in chalkbrood-infected honeybee larvae. J. Chem. Ecol..

[3] Vogt R.G., Riddiford L.M. (1981). Pheromone binding and inactivation by moth antennae. Nature.

[4] Ko H.J., Park T.H. (2008). Enhancement of odorant detection sensitivity by the expression of odorant-binding protein. Biosens. Bioelectron.

[5] Vogt R.G., Rybczynski R., Lerner M.R. (1991). Molecular cloning and sequencing of general odorant-binding proteins GOBP1 and GOBP2 from the tobacco hawk moth *Manduca sexta*: Comparisons with other insect OBPs and their signal peptides. J. Neurosci..

[6] McKenna M.P., Hekmat-Scafe D.S., Gaines P., Carlson J.R. (1994). Putative Drosophila pheromone-binding proteins expressed in a subregion of the olfactory system. J. Biol. Chem..

[7] Steinbrecht R.A. (1998). Odorant-binding proteins: Expression and function. Ann. N. Y. Acad. Sci..

[8] De Barro P.J., Driver F. (1997). Use of RAPD PCR to Distinguish the B Biotype from Other Biotypes of *Bemisia tabaci* (Gennadius) (Hemiptera: Aleyrodidae). Aust. J. Entomol..

[9] Luo C., Jones C., Devine G., Zhang F., Denholm I., Gorman K. (2010). Insecticide resistance in *Bemisia tabaci* biotype Q (Hemiptera: Aleyrodidae) from China. Crop. Prot..

[10] Wang Z., Yan H., Yang Y., Wu Y. (2010). Biotype and insecticide resistance status of the whitefly *Bemisia tabaci* from China. Pest. Manag. Sci..

[11] Denholm I. (1998). Challenges with managing insecticide resistance in agricultural pests, exemplisfied by the whitefly *Bemisia tabaci*. Philos. Trans. R. Soc. B Biol. Sci..

[12] Kessler S.C., Tiedeken E.J., Simcock K.L., Derveau S., Mitchell J., Softley S., Stout J.C., Wright G.A. (2015). Bees prefer foods containing neonicotinoid pesticides. Nature.

[13] Desneux N., Decourtye A., Delpuech J.-M. (2007). The Sublethal Effects of Pesticides on Beneficial Arthropods. Annu. Rev. Entomol..

[14] Horowitz A.R., Kontsedalov S., Khasdan V., Ishaaya I. (2005). Biotypes B and Q of *Bemisia tabaci* and their relevance to neonicotinoid and pyriproxyfen resistance. Arch. Insect Biochem. Physiol..

[15] Whalon M., Mota-Sanchez D., Hollingworth R., Duynslager L. (2019). Arthropod Pesticide Resistance Database.

[16] Brown J.K., Frohlich D.R., Rosell R.C. (1995). The Sweetpotato or Silverleaf Whiteflies: Biotypes of *Bemisia tabaci* or a Species Complex?. Annu. Rev. Entomol..

[17] Costa H.S., Brown J.K., Byrne D.N. (1991). Life History Traits of the Whitefly, *Bemisia tabaci* (Homoptera: Aleyrodidae) on Six Virus-Infected or Healthy Plant Species. Environ. Entomol..

[18] Ohnesorge B., Sharaf N., Allawi T. (2010). Population studies on the tobacco whitefly *Bemisia tabaci* Genn. (Homoptera; Aleyrodidae) during the winter season: I. The spatial distribution on some host plants. J. Appl. Entomol..

[19] Winterhalter P., Rouseff R.L. (2001). Carotenoid-Derived Aroma Compounds: An Introduction. ACS Symp. Ser..

[20] Wang R., Li F., Zhang W., Zhang X., Qu C., Tetreau G., Sun L., Luo C., Zhou J. (2017). Identification and expression profile analysis of odorant binding protein and chemosensory protein genes in *Bemisia tabaci* MED by head transcriptome. PLoS ONE.

[21] Nielsen H., Tsirigos K.D., Brunak S., von Heijne G. (2019). A Brief History of Protein Sorting Prediction. Protein J..

[22] Thompson J.D., Gibson T.J., Plewniak F., Jeanmougin F., Higgins D.G. (1997). The CLUSTAL_X windows interface: Flexible strategies for multiple sequence alignment aided by quality analysis tools. Nucleic Acids Res..

[23] Tamura K., Peterson D., Peterson N., Stecher G., Nei M., Kumar S. (2011). MEGA5: Molecular Evolutionary Genetics Analysis Using Maximum Likelihood, Evolutionary Distance, and Maximum Parsimony Methods. Mol. Biol. Evol..

[24] Deng S., Yin J., Zhong T., Cao Y., Li K. (2011). Function and Immunocytochemical Localization of Two Novel Odorant-Binding Proteins in Olfactory Sensilla of the Scarab Beetle *Holotrichia oblita* Faldermann (Coleoptera: Scarabaeidae). Chem. Senses.

[25] Venthur H., Zhou J., Mutis A., Mella-Herrera R., Larama G., Avila A., Iturriaga-Vasquez P., Faundez-Parraguez M., Alvear M., Quiroz A. (2016). β-Ionone as putative semiochemical suggested by ligand binding on the odorant-binding protein 1 of *Hylamorpha elegans* (Burmeister) and electroantennographic recordings. Entomol. Sci..

[26] Yin J., Feng H., Sun H., Xi J., Cao Y., Li K. (2012). Functional Analysis of General Odorant Binding Protein 2 from the Meadow Moth, *Loxostege sticticalis* L. (Lepidoptera: Pyralidae). PLoS ONE.

[27] Li K., Wang S., Zhang K., Ren L., Ali A., Zhang Y., Zhou J., Guo Y. (2014). Odorant Binding Characteristics of Three Recombinant Odorant Binding Proteins in *Microplitis mediator* (Hymenoptera: Braconidae). J. Chem. Ecol..

[28] Wei D., Ye Z., Gao J., Dong S. (2013). Molecular cloning and functional identification of a Minus-C odorant binding protein from the rice striped stem borer, *Chilo suppressalis* (Lepidoptera: Pyralidae). Acta Entomol. Sin..

[29] Li H., Zhang L., Zhuang S., Ni C., Han B., Shang H. (2013). Interpretation of odorant binding function and mode of general odorant binding protein ASP2 in Chinese honeybee (*Apis cerana cerana*). Sci. Agric. Sin..

[30] Gu S.-H., Wang W.-X., Wang G.-R., Zhang X.-Y., Guo Y.-Y., Zhang Z., Zhou J.-J., Zhang Y.-J. (2011). Functional characterization and immunolocalization of odorant binding protein 1 in the lucerne plant bug, *Adelphocoris lineolatus* (GOEZE). Arch. Insect Biochem. Physiol..

[31] Bailey T.L., Boden M., Buske F.A., Frith M., Grant C.E., Clementi L., Ren J., Li W.W., Noble W.S. (2009). MEME SUITE: Tools for motif discovery and searching. Nucleic Acids Res..

[32] Bailey T.L., Elkan C. (1994). Fitting a mixture model by expectation maximization to discover motifs in bipolymers. Proc. Int. Conf. Intell. Syst. Mol. Biol..

[33] Löbel D., Strotmann J., Jacob M., Breer H. (2001). Identification of a Third Rat Odorant-binding Protein (OBP3). Chem. Senses.

[34] Zhang S., Chen L.-Z., Gu S.-H., Cui J.-J., Gao X.-W., Zhang Y.-J., Guo Y.-Y. (2011). Binding characterization of recombinant odorant-binding proteins from the parasitic wasp, *Microplitis mediator* (Hymenoptera: Braconidae). J. Chem. Ecology.

[35] Congdon R., Muth G., Splittgerber A. (1993). The Binding Interaction of Coomassie Blue with Proteins. Anal. Biochem..

[36] Peitsch M.C., Peitsch M. (1996). ProMod and Swiss-Model: Internet-based tools for automated comparative protein modelling. Biochem. Soc. Trans..

[37] Sali A., Potterton L., Yuan F., Van Vlijmen H., Karplus M. (1995). Evaluation of comparative protein modeling by MODELLER. Proteins Struct. Funct. Bioinform..

[38] Pontius J., Richelle J., Wodak S.J. (1996). Deviations from standard atomic volumes as a quality measure for protein crystal structures. J. Mol. Biol..

[39] Xu P., Atkinson R., Jones D.N., Smith D.P. (2005). Drosophila OBP LUSH is required for activity of pheromone-sensitive neurons. Neuron.

[40] Zwiebel L.J., Takken W. (2004). Olfactory regulation of mosquito-host interactions. Insect Biochem. Mol. Biol..

[41] Laughlin J.D., Ha T.S., Jones D.N.M., Smith D.P. (2008). Activation of pheromone-sensitive neurons is mediated by conformational activation of pheromone-binding protein. Cell.

[42] Chénier J.V.R., Pkilògene B.J.R. (1989). Field responses of certain forest Coleoptera to conifer monoterpenes and ethanol. J. Chem. Ecol..

[43] Cavasotto C.N., Phatak S.S. (2009). Homology modeling in drug discovery: Current trends and applications. Drug Discov. Today.

