# Supplementary material for: Discrimination of Oviposition Deterrent Volatile β-Ionone by Odorant-Binding Proteins 1 and 4 in the Whitefly Bemisia tabaci
Source: Biomolecules. 2019 Oct 3;9(10):563. doi: 10.3390/biom9100563 (PMC6843521; doi:10.3390/biom9100563)
Supplement: Supplementary file 1 [file biomolecules-09-00563-s001.pdf]

## Supplementary Materials

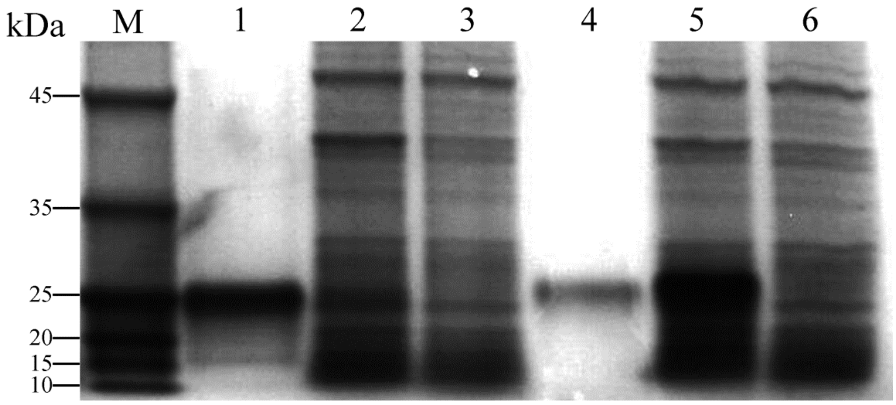


**Figure 1.** Expression and purification of the recombinant OBP1 and OBP4 protein. M: Protein molecular weight; 1: Purified recombinant protein OBP1; 2: Expression products of OBP1 after induction; 3: Bacterial products containing pET30a-OBP1 without induction; 4: Purified recombinant protein OBP4; 5: Expression products of OBP4 after induction; and 6: Bacterial products containing pET30a-OBP4 without induction.

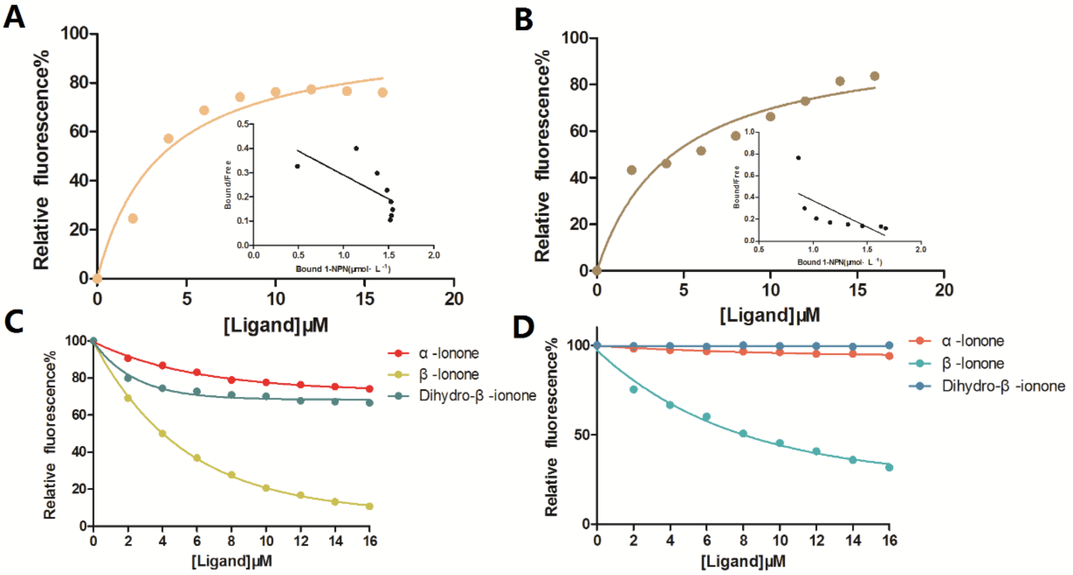


**Figure S2**. Ligand-binding test of OBP1 and OBP4 to ionone. A. Binding of OBP1 and *bis*-ANS; B. Binding of OBP4 and 1-NPN; C. Competitive combining of ionone with *bis*-ANS and OBP1 protein; and D. Competitive binding of ionone with 1-NPN and OBP4 protein.

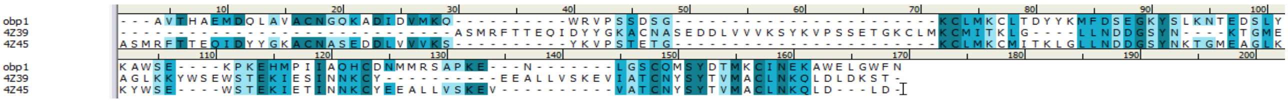


**Figure S3.** Homology alignment of OBP1 with temple.

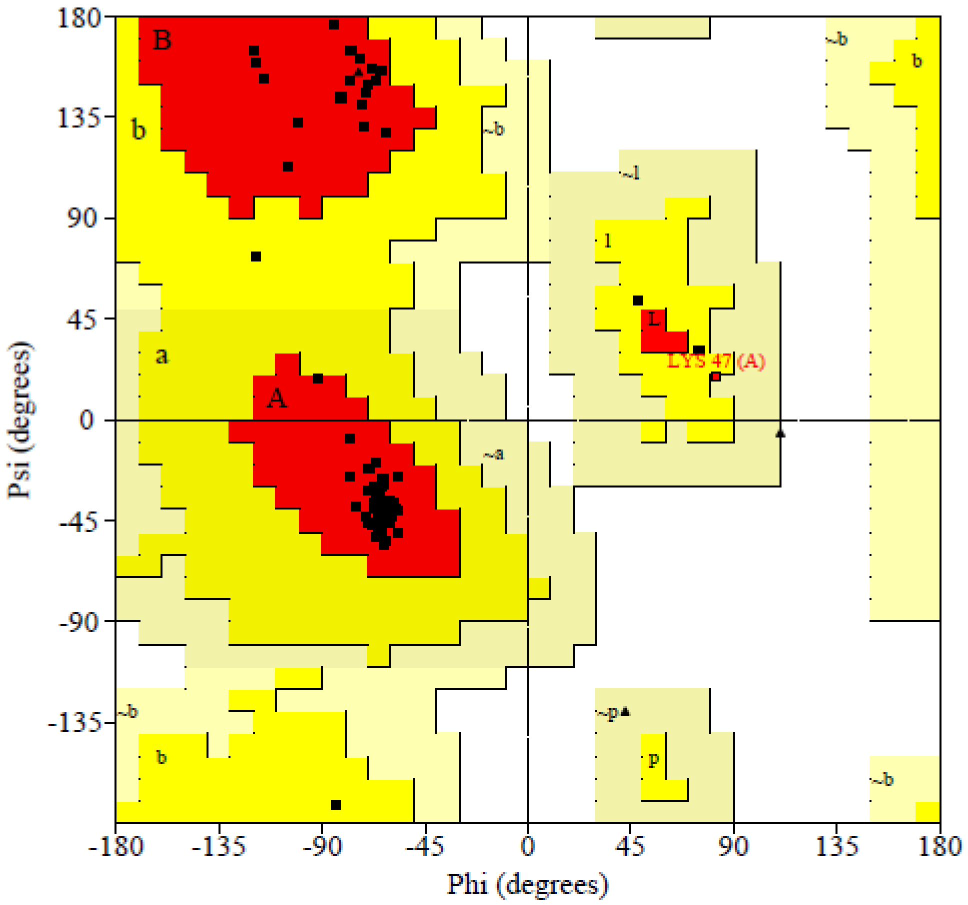


Plot statistics

| | | |
|---|---|---|
| Residues in most favoured regions [A,B,L] | 103 | 96.3% |
| Residues in additional allowed regions [a,b,l,p] | 3 | 2.8% |
| Residues in generously allowed regions [~a,~b,~l,~p] | 1 | 0.9% |
| Residues in disallowed regions | 0 | 0.0% |

**Figure S4.** Ramachandran plot for OBP1.

**Table S1**. NCBI accession numbers for amino acid sequences of OBPs in phylogenetic tree.

| Species | Gene name | Accession number |
|---|---|---|
| *Bemisia tabaci Q* | Btab_Q_OBP1 | |
| | Btab_Q_OBP4 | |
| *Bemisia tabaci B* | Btab_B_OBP1 | |
| | Btab_B_OBP4 | |
| *Acyrthosiphon pisum* | Apis_OBP | ACI30694.1 |
| | Apis_OBP11 | XP_008178459.1 |
| *Megoura viciae* | Mvic_OBP2 | CAR85651.1 |
| | Mvic_OBP3 | AXE72019.1 |
| *Daktulosphaira vitifoliae* | Dvit_OBP3 | ARO50002.1 |
| *Brevicoryne brassicae* | Bbra_OBP3 | AEX65667.1 |
| *Episyrphus balteatus* | Ebal_OBP3 | AEX65666.1 |
| *Harmonia axyridis* | Haxy_OBP3 | AEX65665.1 |
| *Diaphorina citri* | Dcit_OBP1 | ARR95844.1 |
| *Nilaparvata lugens* | Nlug_OBP1 | ASL05033.1 |
| *Aphis gossypii* | Agos_OBP | ACI30678.1 |
| *Laodelphax striatella* | Lstr_OBP4 | AGZ04923.1 |
| *Pterocomma salicis* | Psal_OBP2 | CAR85661.1 |
| *Aphis glycines* | Agly_OBP2 | AHJ80888.1 |
| *Aphis craccivora* | Acra_OBP | CAR85658.1 |
| *Holotrichia oblita* | Hobl_OBP1 | ACX32050.2 |
| | Hobl_OBP2 | ACX32049.2 |
| *Hylamorpha elegans* | Hele_OBP1 | AMR98353.1 |
| *Loxostege sticticalis* | Lsti_OBP2 | ABY75632.1 |
| *Chilo suppressalis* | Csup_OBP1 | AGK24577.1 |
| *Apis cerana* | Acer_OBP2 | ABD97844.2 |
| *Adelphocoris lineolatus* | Alin_OBP1 | ACZ58027.1 |
| *Microplitis mediator* | Mmed_OBP8 | AEF14409.1 |
| | Mmed_OBP10 | AEO27860.1 |

**Table S2**. Binding affinities of odorants to OBP1 and OBP4.

| Proteins | Odorants | $IC_{50}$ | $K_i$ |
|---|---|---|---|
| | α-Ionone | 40.14 | 28.67 |
| OBP1 | β-ionone | 7.16 | 5.15 |
| | Dihydro-β-ionone | — | — |
| | α-Ionone | 21.10 | 14.86 |
| OBP4 | β-ionone | 5.14 | 3.62 |
| | Dihydro-β-ionone | — | — |

$IC_{50}$ exceeded 50 mM are represented as "—".
